# Interaction between Rsp5-dependent ubiquitination and trehalose production during *Cryptococcus neoformans* temperature stress adaptation

**DOI:** 10.1128/msphere.00212-26

**Published:** 2026-05-20

**Authors:** Alejandro L. Antonia, Lukas M. du Plooy, Siobhan R. Duffy, Jeffrey Kuhn, Erik J. Soderblom, J. Andrew Alspaugh

**Affiliations:** 1 Department of Medicine, Duke University School of Medicine, Durham, North Carolina, USA; 2 Department of Molecular Genetics and Microbiology, Duke University School of Medicine, Durham, North Carolina, USA; 3 Proteomics and Metabolomics Core Facility, Duke University School of Medicine, Durham, North Carolina, USA; 4 Department of Cell Biology, Duke University School of Medicine, Durham, North Carolina, USA; University of Wisconsin-Madison, Madison, Wisconsin, USA

**Keywords:** fungal pathogenesis, *Cryptococcus neoformans*, trehalose, ubiquitination, osmotic stress, stress response, temperature stress

## Abstract

Microorganisms, including fungi, adapt to profound changes in their local environment during human infections. After exposure to high temperature and other stress conditions, the opportunistic fungal pathogen *Cryptococcus neoformans* enacts changes in metabolism, cell wall structure, and transmembrane transport that allow it to survive and proliferate in a mammalian host. This stress response program is regulated by the HECT E3-ubiquitin ligase Rsp5, which is required for growth at high salinity, pH, and temperature. However, the complete set of Rsp5 substrates that direct these molecular changes remains incompletely understood. Here, we demonstrate that *C. neoformans* Rsp5 confers increased tolerance to temperature and salt stress in part through regulation of the trehalose biosynthesis pathway. Two enzymes in the trehalose biosynthesis pathway, Tps1 and Tps2, are differentially ubiquitinated by Rsp5 after exposure to stress conditions. We directly measured trehalose production after exposure to high temperature and found that a *C. neoformans* strain lacking Rsp5 is unable to induce trehalose production. Quantitative proteomic analysis of the *C. neoformans* response to high salinity identified Rsp5-dependent and independent adaptations to osmotic stress, and Rsp5-dependent ubiquitination does not alter the abundance of Tps1 or Tps2. These results suggest that regulation of trehalose biosynthesis is one of the cellular mechanisms by which Rsp5-dependent ubiquitination in *C. neoformans* facilitates survival in response to stressors encountered in the human infection environment.

**IMPORTANCE:**

*Cryptococcus neoformans* is an opportunistic fungal pathogen that kills over 180,000 people every year, with few effective treatment options. As a yeast that normally lives in the environment, *C. neoformans* has to survive large changes in its physical environment, including elevated body temperature, which causes human infections. Here, we show how *C. neoformans* uses a protein modification to regulate production of a fungus-specific metabolic pathway important for survival at human body temperature. Unraveling how environmental fungi tolerate and survive temperature and other stressors will help us to understand how they cause disease and identify new and better ways to treat these deadly infections.

## INTRODUCTION

Microorganisms often encounter drastic shifts in their physical environment, requiring them to alter their metabolism and cellular structures. For the environmental and pathogenic yeast *Cryptococcus neoformans,* the ability to successfully navigate the transition from surviving in soil and trees to a mammalian host allows this microorganism to cause disease, resulting in over 180,000 deaths annually, primarily in immunocompromised individuals (1–3). In response to stressors in human hosts such as elevated temperature and pH, *C. neoformans* undergoes profound molecular adaptations that allow it to survive and proliferate in this hostile environment (4–7).

Host-derived stress adaptations are coordinated by several central signaling pathways. These allow for survival at elevated temperatures, variations in pH, and in the presence of cell wall stressors (8–11). For example, the *C. neoformans* high-osmolarity glycerol (Hog) response pathway consists of a two-component system in which membrane-bound proteins sense environmental changes and activate an intracellular kinase signaling cascade that allows the yeast to tolerate cell wall, high temperature, and osmotic stressors (12, 13). Recent advances have also highlighted that post-translational modifications regulate *C. neoformans* stress adaptation. For example, in response to infection-related stress conditions, targeted protein ubiquitination can direct alterations in cell membrane structure through inositol sphingolipid biosynthesis and cell wall structure through chitin production (14, 15). Ubiquitin is a 76-amino acid moiety that is post-translationally covalently linked to specific lysine residues in target proteins. The ubiquitin cascade consists of E1 activating enzymes, E2 conjugating enzymes, and E3 ligases. The E3 ligases are the most numerous and diverse components of this cascade and thus help determine ubiquitin target substrate specificity.

The E3-ubiquitin ligase Rsp5 in *C. neoformans* is required for growth under host-relevant stress conditions. When the corresponding gene is deleted, this pathogen becomes avirulent in animal models of infection (15). We have previously demonstrated that under high salinity and alkaline growth conditions, Rsp5 differentially ubiquitinates over 180 proteins. A detailed analysis of proteins with known Rsp5-binding motifs highlights the pleiotropic activity of this enzyme, with its substrates involved in transmembrane transport, cell wall structure, and transcriptional responses to temperature stress (15). Here, a comparative analysis between substrates ubiquitinated differentially between the high salinity and alkaline stress conditions identified a novel interaction between Rsp5 and trehalose biosynthesis.

Trehalose is a disaccharide with a biosynthetic pathway conserved across the fungal kingdom, including pathogenic fungi, which is involved in the maintenance of cell wall structure, tolerance to temperature stress, sexual differentiation, and as an alternative energy source (16, 17). For these reasons, disruption of the *C. neoformans* trehalose biosynthesis pathway leads to impaired virulence (18). The biosynthesis of trehalose in fungi, including *C. neoformans,* canonically involves two enzymes: (i) Tps1 (alpha,alpha-trehalose-phosphate synthase), which converts glucose-6-phosphate to trehalose-6-phosphate, and (ii) Tps2 (trehalose-6-phosphate phosphatase), which converts trehalose-6-phosphate to trehalose. In *C. neoformans,* trehalose homeostasis is also maintained by degradation through two trehalase enzymes (Nth1 and Nth2) (17). Because trehalose biosynthesis is required for fungal virulence but is not present in mammals, it represents an enticing novel target for anti-fungal development (19). However, the precise mechanisms of regulation of the trehalose biosynthesis pathway by post-translational ubiquitination, especially with regard to microbial pathogens, have yet to be fully described.

In this study, we explore the relationship between Rsp5-mediated ubiquitination and trehalose biosynthesis. An unbiased analysis of Rsp5 target proteins identified trehalose biosynthesis proteins as possible targets of ubiquitination mediating microbial response to host-derived stresses. By mass-spectrometry-based direct protein quantification, we show that Rsp5 does not alter the total protein abundance of the known components of the trehalose biosynthesis pathway. Finally, we demonstrate that deletion of Rsp5 impairs *C. neoformans* induction of trehalose production in response to temperature stress, suggesting that Rsp5-dependent temperature sensitivity is, in part, mediated by osmotic tolerance. Together, these studies are the first demonstration of post-translational modifications regulating trehalose biosynthesis in pathogenic fungi.

## RESULTS

### *C. neoformans* Rsp5 ubiquitinates effectors of microbial stress response pathways, including trehalose biosynthesis

*C. neoformans* is an opportunistic fungal pathogen that needs to adapt to significant changes in its biological and abiotic environment when moving between its environmental niche and the human host. This is, in part, achieved through changes in ubiquitination. We have previously demonstrated that the HECT E3-ubiquitin ligase Rsp5 is required for stress adaptation and virulence (15). Prior screens to identify changes in ubiquitination of specific substrates under high salt conditions and alkaline stress conditions led to mechanistic studies confirming that Rsp5 regulates both nucleobase transport and Rim101 pathway activation as two distinct downstream mechanisms of adaptation to host-stress conditions (15, 20). However, it remains unclear whether Rsp5-mediated changes in ubiquitination are a common response to host-derived stress in general or specific to individual stressors.

**Fig 1:**
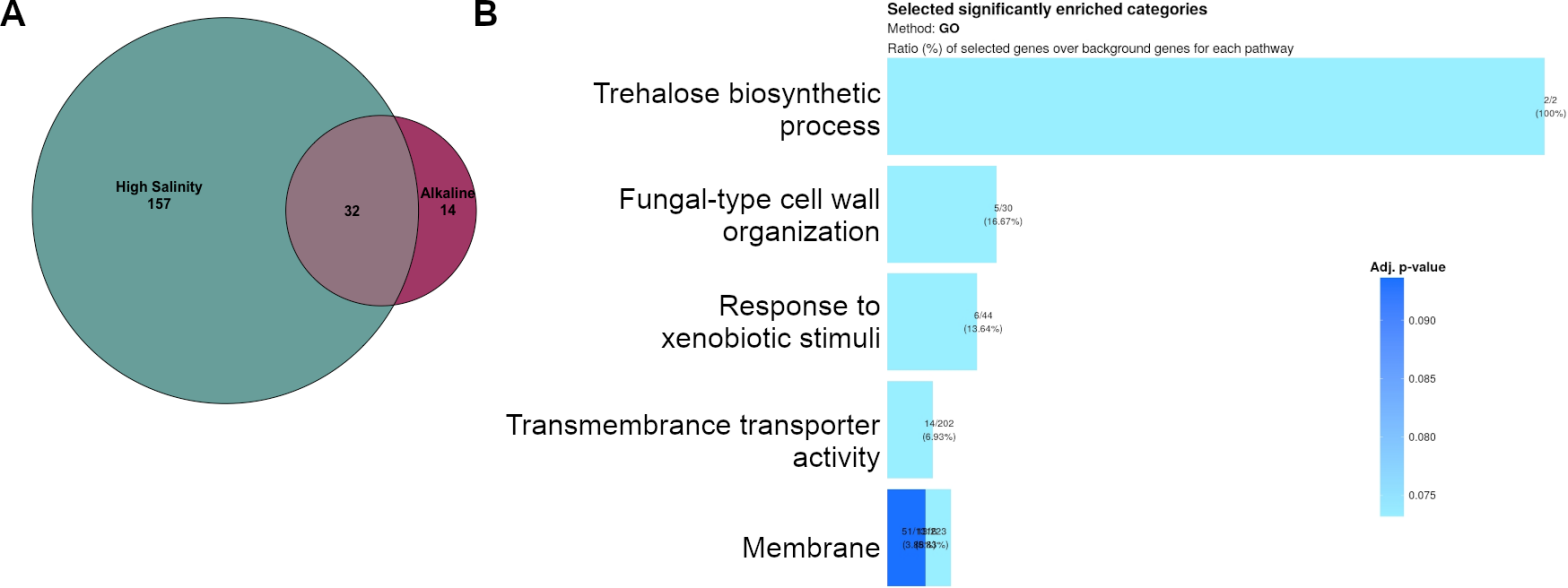
*C. neoformans* E3-ubiquitin ligase Rsp5 ubiquitinates distinct substrates in response to specific stress conditions, including the trehalose biosynthesis pathway. (**A**) Comparison of differential ubiquitination of likely Rsp5 substrates between host-derived stress conditions. *C. neoformans* was exposed to either high salinity (NaCl 1.5M) or alkaline pH (pH 8.15) for 1 h prior to measuring ubiquitination of substrates by LC/MS-MS present in a wild type but not in the *rsp5*∆ strain. (**B**) Gene ontology analysis identified the trehalose biosynthesis pathway as significantly enriched among Rsp5 substrates after exposure to osmotic stress. The substrates exclusively ubiquitinated by Rsp5 under high salinity stress were analyzed for enrichment of assigned gene ontology terms using the FungiFun3 webserver.

A comparison of differentially ubiquitinated substrates between the conditions of alkaline stress and high salinity suggests that Rsp5 has a set of substrates that are generally altered under multiple host-stress conditions and a set of substrates that are specific to unique individual stress conditions. To test this hypothesis, we determined the differential ubiquitination of substrates by Rsp5 after incubating both the *C. neoformans* wild type and the *rsp5*∆ mutant strain under high salinity (NaCl 1.5M) or alkaline (pH 8.15) conditions for 1 h, as previously described (15, 20). Ubiquitinated proteins were specifically enriched in these lysates by immunoaffinity using an antibody that recognizes specific modifications of previously ubiquitinated lysine residues after trypsin digestion (the ubiquitin “stump”). We used a stringent threshold for enrichment of a 5-fold increase in ubiquitination between the wild type and *rsp5*∆ strains to avoid interpreting false positive substrates. This identified 46 potential *C. neoformans* Rsp5 substrates for ubiquitination under alkaline stress and 189 substrates under high salinity stress. Of these, 32 substrates were enriched as likely Rsp5 substrates under both stress conditions (Fig. 1A).

To identify novel mechanisms of stressor-specific adaptations, we then performed gene ontology (GO) analysis for the set of proteins unique to either alkaline stress (no significant GO term enrichment observed) or high salinity conditions. As previously characterized, the high salinity-specific Rsp5 substrates were enriched for proteins predicted to mediate cellular processes, including transmembrane transport and cell membrane integrity (15, 20). However, Rsp5-dependent ubiquitination was also observed for Tps1 (CNAG_05292) and Tps2 (CNAG_03765), two enzymes involved in the biosynthesis of the osmotically stabilizing sugar trehalose, specifically under high salt stress (Fig. 1B).

This result suggested a novel link between Rsp5-dependent ubiquitination and trehalose biosynthesis, two central components of *C. neoformans* adaptation to host stress and virulence (15, 18–20). We therefore hypothesized that Rsp5-mediated ubiquitination would change the abundance or function of trehalose biosynthesis enzymes under host stress conditions.

### The *C. neoformans* proteomic response to high salinity includes Rsp5-dependent and independent changes

**Fig 2:**
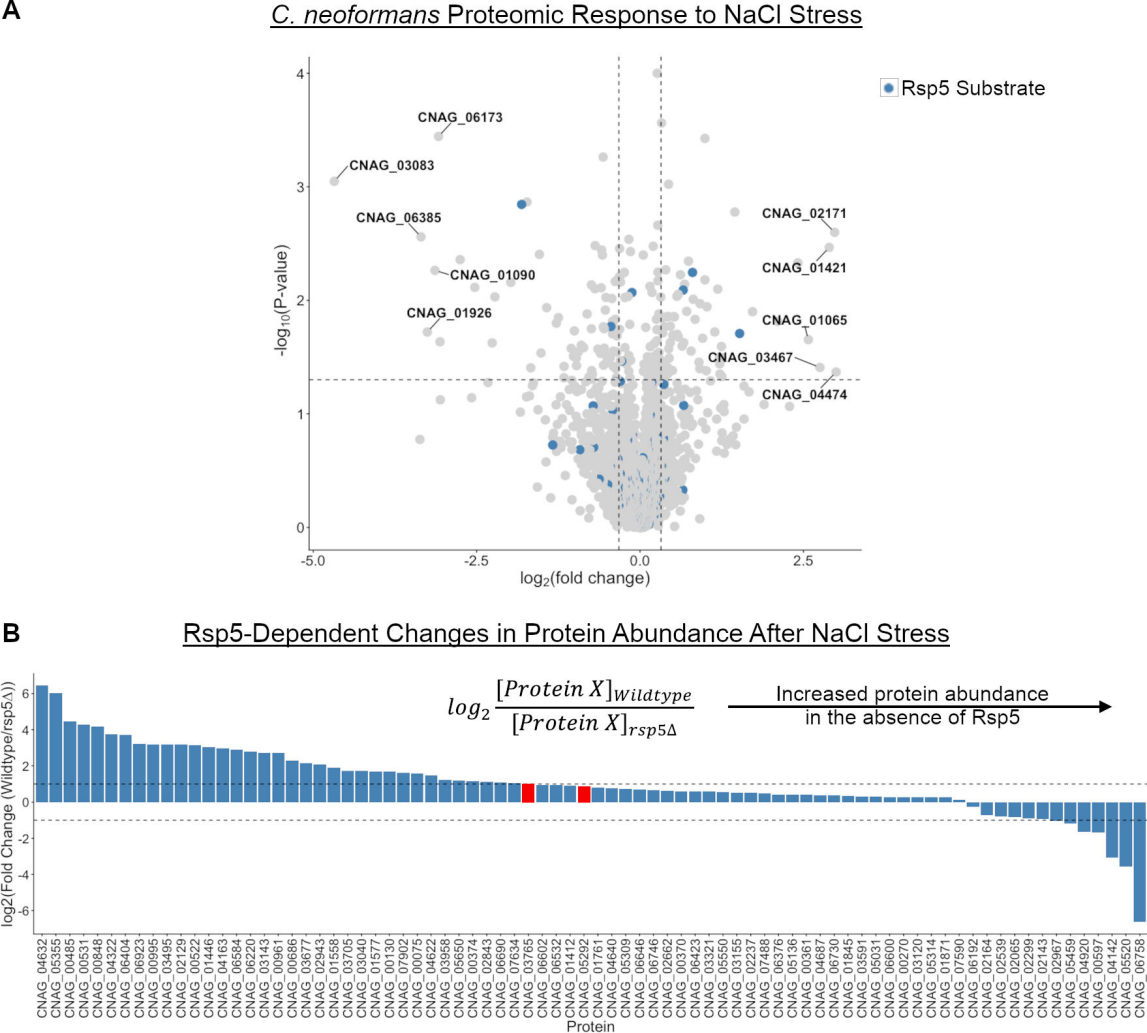
The *C. neoformans* proteome in response to osmotic stress includes both Rsp5-dependent and Rsp5-independent changes. (**A**) Volcano plot demonstrating *C. neoformans* changes in proteome in response to osmotic stress. Blue dots represent previously identified substrates of Rsp5 ubiquitination. The gene names of the five most highly increased and decreased genes are indicated by text and are described in further detail in Table 1. Wild-type *C. neoformans* was incubated in liquid YPD supplemented with 1.5 M NaCl for 1 h prior to protein harvest. Protein quantification was performed by liquid chromatography-tandem mass spectrometry (LC-MS/MS). (**B**) Rsp5-dependent changes in protein abundance after osmotic stress with 1.5 M NaCl were determined by calculating the fold change between wild-type and *rsp5*∆ strains of *C. neoformans*. Absolute fold change values less than 1 [log_2_(fold change) < 0] represent an increase in protein abundance in the absence of Rsp5. Only proteins with a statistically significant (*P* < 0.05) change are plotted. The dashed horizontal line represents an absolute fold change cutoff of 2.0. Red bars represent known components of the trehalose biosynthesis pathway (Tps1 and Tps2).

To specifically define the role of osmotic stress and Rsp5 in *C. neoformans* protein homeostasis, we performed proteomics to characterize whole-cell changes in protein abundance after brief exposure to high salinity stress. We incubated the *C. neoformans* wild type and *rsp5*∆ strains in high salinity (NaCl 1.5M) osmotic stress for 1 h, and total protein lysates were harvested for quantitative mass spectrometry analysis to analyze total changes in the proteome. In the wild-type strain, 128 proteins demonstrated a statistically significant alteration in total abundance after exposure to high salinity stress (Fig. 2A; Table S1). Proteins were deemed to be significantly changed if they were identified by at least two unique peptide precursors, had an absolute fold change of 1.25 ($\frac{{\left[protein\;X\right]}_{YPD}}{{\left[protein\;X\right]}_{YPD+1.5M\;NaCl}}$), and had an unadjusted *P*-value of less than 0.05. Among these 128 proteins, five were previously identified as possible Rsp5 substrates (Fig. 2A) (15). Of these five proteins, two have predicted roles in nucleobase transport (CNAG_00597 and CNAG_04632), two have predicted roles in cellular metabolism (CNAG_03040 and CNAG_04920), and one has no identifiable function based on sequence and domain analysis (CNAG_03415). Interestingly, of the two predicted amino acid transporters, CNAG_04632 (Nbt1) was previously demonstrated to have altered localization and abundance in a Rsp5-dependent manner (15), and CNAG_00597 is transcriptionally upregulated in response to osmotic stress (21).

Of the remaining proteins with altered abundance after osmotic stress, gene ontology and pathway analysis did not identify any significantly enriched cellular processes after osmotic stress. Notably, although both Tps1 and Tps2 are each individually required for *C. neoformans* thermotolerance and stress responses (18), neither protein demonstrated a measurable change in abundance in the wild-type strain in response to this condition. However, assigned functional terms for individual proteins with altered abundance in this assay highlighted other proteins with known roles in *C. neoformans* temperature and osmotic tolerance. For example, the Msb2 protein is increased in abundance by 7.4-fold (*P* = 0.0003) after exposure to NaCl stress (Table 1). The homolog of this protein in *Saccharomyces cerevisiae* is a membrane sensor of osmotic stress that activates a kinase signaling cascade, which promotes osmotic stress tolerance (22). Similarly, *C. neoformans* Msb2 contributes to osmotic stress tolerance with other components of the HOG signaling pathway, and its deletion leads to impaired fungal survival in a murine pulmonary infection model (13). Therefore, an analysis of the *C. neoformans* proteomic response to high salinity identifies both Rsp5-dependent and independent mechanisms of osmotic stress tolerance.

**TABLE 1:** Top differentially expressed proteins by *C. neoformans* in response to high salinity stress^*a*^

Gene ID	Gene name	log2(FC)	*P*-value	Computed GO functions
CNAG_04474	N/A^*b*^	3.00	4.28E−02	Transmembrane transporter activity
CNAG_02171	N/A	2.98	2.52E−03	Damaged DNA binding
CNAG_01421	MSB2	2.90	3.42E−03	Osmosensor activity
CNAG_03467	N/A	2.75	3.90E−02	N/A
CNAG_01065	N/A	2.58	2.22E−02	Hydrolase activity
CNAG_06173	N/A	−3.08	3.60E−04	Hydrolase activity
CNAG_01090	N/A	−3.14	5.45E−03	Catalytic activity; hydrolase activity
CNAG_01926	PEX5	−3.25	1.90E−02	Protein binding
CNAG_06385	N/A	−3.35	2.76E−03	Protein binding
CNAG_03083	N/A	−4.68	8.95E−04	Nutrient reservoir activity

^*a*^ The top five highest and lowest differentially expressed proteins after osmotic stress include known components of the *C. neoformans* osmotic stress response, such as Msb2. proteins ranked by the fold change (FC) of wild-type *C. neoformans* expression under NaCl stress compared to the control condition.

^*b*^ “N/A” indicates “not applicable”.

### Rsp5 does not alter the total protein abundance of trehalose biosynthesis enzymes in response to osmotic stress

In addition to defining proteomic changes in the wild-type strain after osmotic stress, we also characterized total proteomic changes in this stress condition between the wild-type and *rsp5*Δ mutant strain. In this way, we would be able to identify osmotic and Rsp5-dependent changes in protein abundance that may not necessarily be reflected simply in the wild-type strain. Rsp5-dependent changes in protein abundance in response to osmotic stress were determined by comparing the fold change between the wild-type *C. neoformans* strain and the *rsp5*∆ mutant strain ($\frac{{\left[protein\;X\right]}_{wild\;type}}{{\left[protein\;X\right]}_{rsp5\Delta }}$) when exposed to high salinity (NaCl 1.5M) and are reported for the known Rsp5 substrates in Table S2. Notably, the trehalose-degrading enzymes, Nth1 and Nth2, were not identified as likely Rsp5 substrates (15) and are not significantly changed in abundance after NaCl stress in the wild-type or *rsp5*∆ conditions (Fig. 2A; Table S2). In total, seven of the known likely Rsp5 substrate proteins displayed at least a 2.0-fold increase in abundance in the absence of Rsp5 (Fig. 2B), consistent with ubiquitin-mediated proteasomal degradation during osmotic stress. In contrast, Tps1 and Tps2 demonstrated a modest increase in protein abundance in the absence of Rsp5 (1.84-fold and 2.02-fold, respectively). Therefore, this result suggests that the Rsp5-mediated alterations of the Tps1 and Tps2 trehalose biosynthesis enzymes result in proteasome-independent cellular processes in response to osmotic stress.

### Rsp5 ubiquitinates lysine residues distinct from the catalytic regions of Tps1 (lysine-228) and Tps2 (lysine-78)

**Fig 3:**
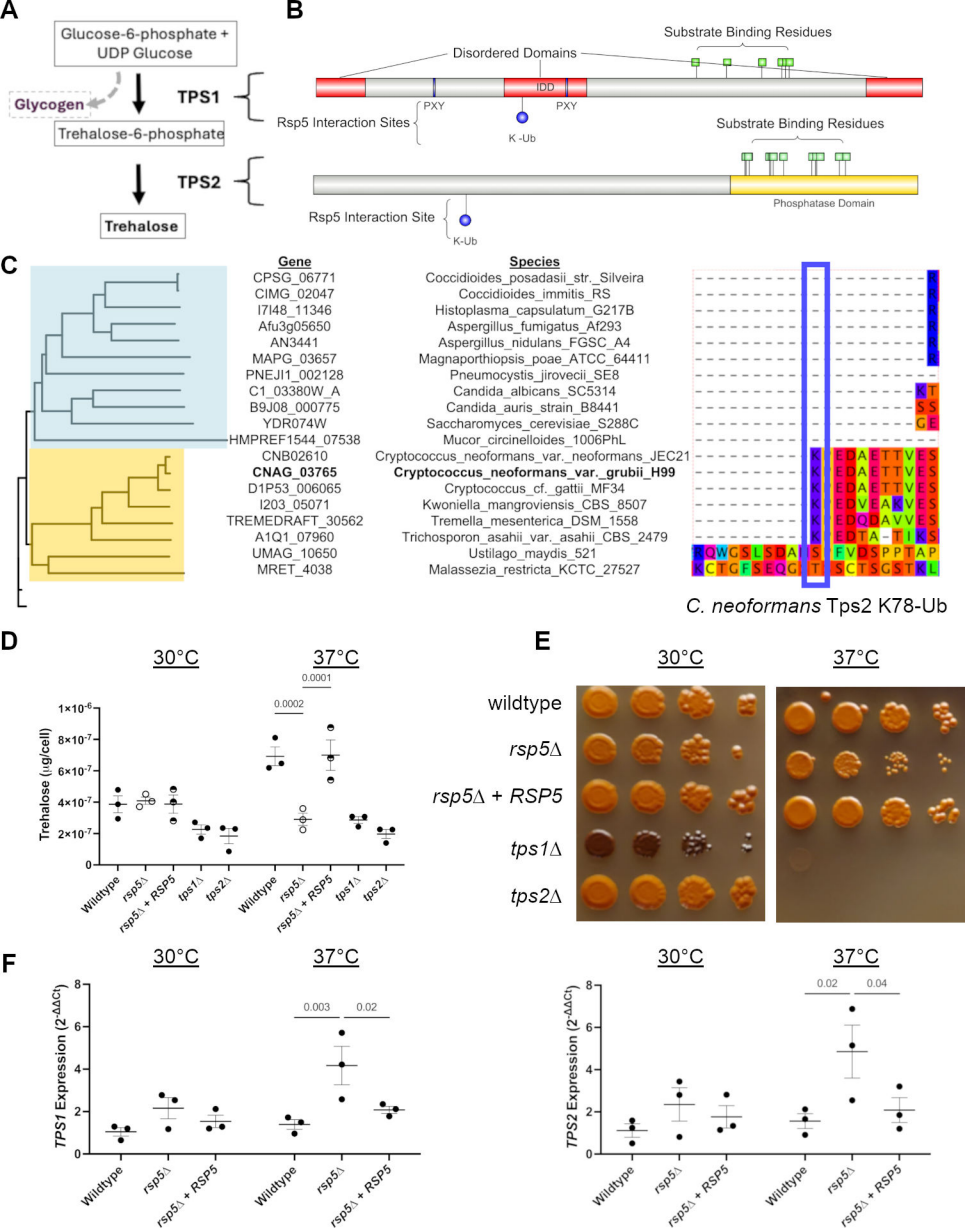
*C. neoformans* induction of trehalose production in response to temperature stress is Rsp5-dependent. (**A**) Simplified schema of trehalose biosynthesis, including the enzymes alpha,alpha-trehalose-phosphate synthase (Tps1) and trehalose-6-phosphate phosphatase (Tps2). (**B**) Domain diagram of *C. neoformans* Tps1 and Tps2. Rsp5 interaction sites are highlighted in blue, including putative PXY binding motifs and the ubiquitination sites K228 on Tps1 and K78 on Tps2, identified by LC-MS-MS after exposure to high salinity stress. Known enzymatic residues are highlighted by green squares. (**C**) The Tps2 K78 residue ubiquitinated by Rsp5 is in a basidiomycete-specific segment of the protein. Tps2 homologs were identified by BlastP with a search limited to a manually curated set of representative reference organisms. The phylogeny and multisequence alignment were generated by the Clustal algorithm. Clades that included proteins with known alternative functions (such as Tps1) were collapsed in the presented phylogeny. (**D**) After temperature stress, *C. neoformans* upregulation of trehalose production is Rsp5 dependent. Trehalose quantification was determined after 8 h at the indicated temperature and normalized per individual cell as quantified by hemocytometer. Mean and standard error of the mean plotted from three biological replicates. *P*-values are calculated by two-way ANOVA with Tukey’s post-hoc test. (**E**) Loss of Rsp5 does not result in hyperaccumulation of glycogen. Each strain was diluted to a starting OD of 1.0 with 5 serial 10× dilutions prior to pinning onto YPD agar plates and incubating at the indicated temperature for 72 h. Iodine vapor was used to stain each plate for glycogen, with violet color representing a greater concentration of carbohydrates, including glycogen. Representative images shown from three separate biological replicates are shown. (**F**) *TPS1* and *TPS2* gene expression is increased in the *rsp5*∆ mutant strain. *C. neoformans* wild type, *rsp5*∆, and *rsp5*∆ + *RSP5* were incubated at 37°C for 4 h prior to mRNA harvest. *TPS1* and *TPS2* expression was measured by qRT-PCR with gene-specific primers designed to overlap an exon-junction. The relative expression was determined by the ∆∆ct method relative to the housekeeping gene *GPD1*. Mean and standard error of the mean plotted from three biological replicates. *P*-values calculated by two-way ANOVA with Tukey’s *post-hoc* test.

Our prior differential ubiquitination assay for identifying potential Rsp5 substrates used liquid chromatography-tandem mass spectrometry (LC-MS/MS) to quantify ubiquitinated proteins present after stress conditions in either the *C. neoformans* wild-type or *rsp5*∆ strain after enriching with an antibody that recognizes the remnant ubiquitin stump after trypsin digestion (15, 20). One advantage of this approach is that the remnant of the anti-ubiquitin antibody can be resolved by LC-MS-MS, thus specifically identifying those residues that are likely ubiquitinated by Rsp5. Representative spectra for the ubiquitinated peptides identified within Tps1 (K-228) and Tps2 (K-78) are shown with the quantitative peptide counts for the wild-type and *rsp5*∆ conditions (Fig. S1). For both of these enzymes, the ubiquitination sites are distant from known catalytic or functional domains (Fig. 3B) (19, 23). Interestingly, previous investigation has demonstrated that the location of the Tps1 K228 residue is located within an internal disordered domain (IDD) of uncertain functional significance, but which is exclusively present in Tps1 orthologs in basidiomycete fungi (19). Similarly, the Rsp5 ubiquitinated residue on Tps2 (K78) is located in a region without structural data. Like Tps1, Tps2 sequences from representative fungi distinctly cluster between the ascomycete and basidiomycete phyla, and the basidiomycete Tps2 proteins contain several internal regions that are not present in ascomycetes. Similar to Tps1, the ubiquitinated lysine on Tps2 (K78) is located in one such basidiomycete-specific region (Fig. 3C). This suggests that Rsp5-mediated ubiquitination of these trehalose biosynthesis enzymes may be a unique phenomenon in this fungal phylum.

### *C. neoformans t*rehalose production after temperature stress is Rsp5-dependent

We then sought to directly measure how Rsp5 alters *C. neoformans* production of trehalose after temperature stress, the most well-studied condition in which trehalose is cytoprotective. In the trehalose biosynthesis pathway, glucose-6-phosphate is first converted to trehalose-6-phosphate, which is in turn converted to trehalose. Mutations in either of the *tps1*Δ or *tps2*Δ strains result in failed trehalose production. Specifically, for the *tps1*Δ strain, in which the microbe is unable to perform the first step in this enzymatic pathway, the input glucose-6-phosphate is shunted to result in increased glycogen production (summarized in Fig. 3A). To quantify trehalose production, we first incubated the wild-type, *tps1*Δ, *tps2*Δ, and *rsp5*Δ strains in YPD medium at 30°C and 37°C for 8 h. We then used a biochemical assay to measure intracellular trehalose. Previous investigators have used this assay in several fungal species, including *S. cerevisiae, A. fumigatus,* and *C. neoformans,* to demonstrate increased intracellular trehalose levels after temperature shift as part of a thermal adaptive response (17, 24–26). As previously noted, the wild-type strain produced over twice as much trehalose per cell at 37°C compared to 30°C. Also, as expected, the *tps1*Δ and *tps2*Δ strains failed to produce significantly measurable amounts of trehalose at either temperature. Although at 30°C, the *rsp5*∆ mutant produces wild-type levels of trehalose, at the 37°C stress condition, the *rsp5*Δ mutant produces significantly less trehalose than the wild-type strain (2.9 × 10^−7^ µg/cell vs. 6.9 × 10^−7^ µg/cell; *P* = 0.0002), which is similar to the trehalose biosynthesis enzyme mutants (Fig. 3D). The very low levels of trehalose measured in all mutant strains at the higher temperature (near the limit of assay detection) suggest that additional, related sugars did not accumulate in these strains that would potentially confound data interpretation. We confirmed that the failed induction of trehalose production at high temperature was an Rsp5-dependent effect by demonstrating complete restoration of trehalose production by complementation of *RSP5* into the *rsp5*Δ mutant background (Fig. 3D).

We also measured trehalose production in response to NaCl stress and found that all strains tested (wild type, *rsp5*∆, and *rsp5*∆*+RSP5*) demonstrated a marked decrease in trehalose levels after exposure to NaCl (1.5M) for 8 h (Fig. S2). While there appeared to be a trend toward lower relative trehalose production in the *rsp5*∆ strain (mean 79.95 μg/mL) compared to wild type (mean 255.39 μg/mL) when normalized to final culture concentration, this difference was not statistically significant, likely due to the very low total cellular trehalose levels in all strains in this stress condition (near the limit of assay detection).

Although both the *tps1*Δ and *tps2*Δ strains are similarly defective in trehalose production, individual defects in each of these enzymes can be distinguished by measuring glycogen accumulation. Glycogen levels are uniquely elevated in the *tps1*Δ mutant due to metabolic shunting of hyper-accumulated glucose-6-phosphate toward glycogen synthesis (Fig. 3E). To test whether the trehalose defect of the *rsp5*Δ mutant could be attributed to reduced activity of one or both trehalose biosynthetic enzymes, we performed colorimetric iodine staining as a marker of total carbohydrate content, which has previously been used as a surrogate for glycogen accumulation (27). Predictably, the *tps1*Δ strain demonstrated enhanced iodine staining, suggesting increased glycogen accumulation. In contrast, the *rsp5*∆ strain did not accumulate increased glycogen at either 30°C or 37°C (Fig. 3E), suggesting that the Tps1 enzyme retains sufficient activity in the absence of Rsp5 to prevent hyperaccumulation of glycogen despite the notable decrease in overall trehalose production.

### Expression of the *TPS1* and *TPS2* genes increases in the absence of Rsp5

Because the diverse Rsp5 substrates include regulators and transcription factors of multiple stress response pathways (15, 20), we next tested whether the Rsp5-dependent decrease in total trehalose is due to changes in *TPS1* or *TPS2* gene expression. The *C. neoformans* wildtype, *rsp5*∆, or *rsp5*∆+*RSP5* were shifted to either 30°C or 37°C for 4 h prior to harvesting mRNA for gene expression analysis by quantitative real-time polymerase chain reaction (qRT-PCR). After this temperature stress, the wild-type strain did not induce expression of either the *TPS1* or *TPS2* genes despite increased trehalose production at 37°C, suggesting transcription-independent mechanisms of temperature-regulated trehalose induction. In contrast, the *C. neoformans rsp5*Δ mutant displayed a significant upregulation of both *TPS1* and *TPS2* transcripts compared to both the wild-type and *rsp5*Δ + *RSP5* complemented strain (Fig. 3F). Therefore, the decreased trehalose production in the *rsp5*Δ mutant is not due to decreased transcription of these genes. In contrast, the induction of *TPS1* and *TPS2* in the absence of Rsp5 suggests compensatory upregulation of trehalose biosynthesis genes in response to impaired total trehalose production.

### *C. neoformans* Rsp5-dependent temperature sensitivity is partially due to impaired osmotic tolerance

**Fig 4:**
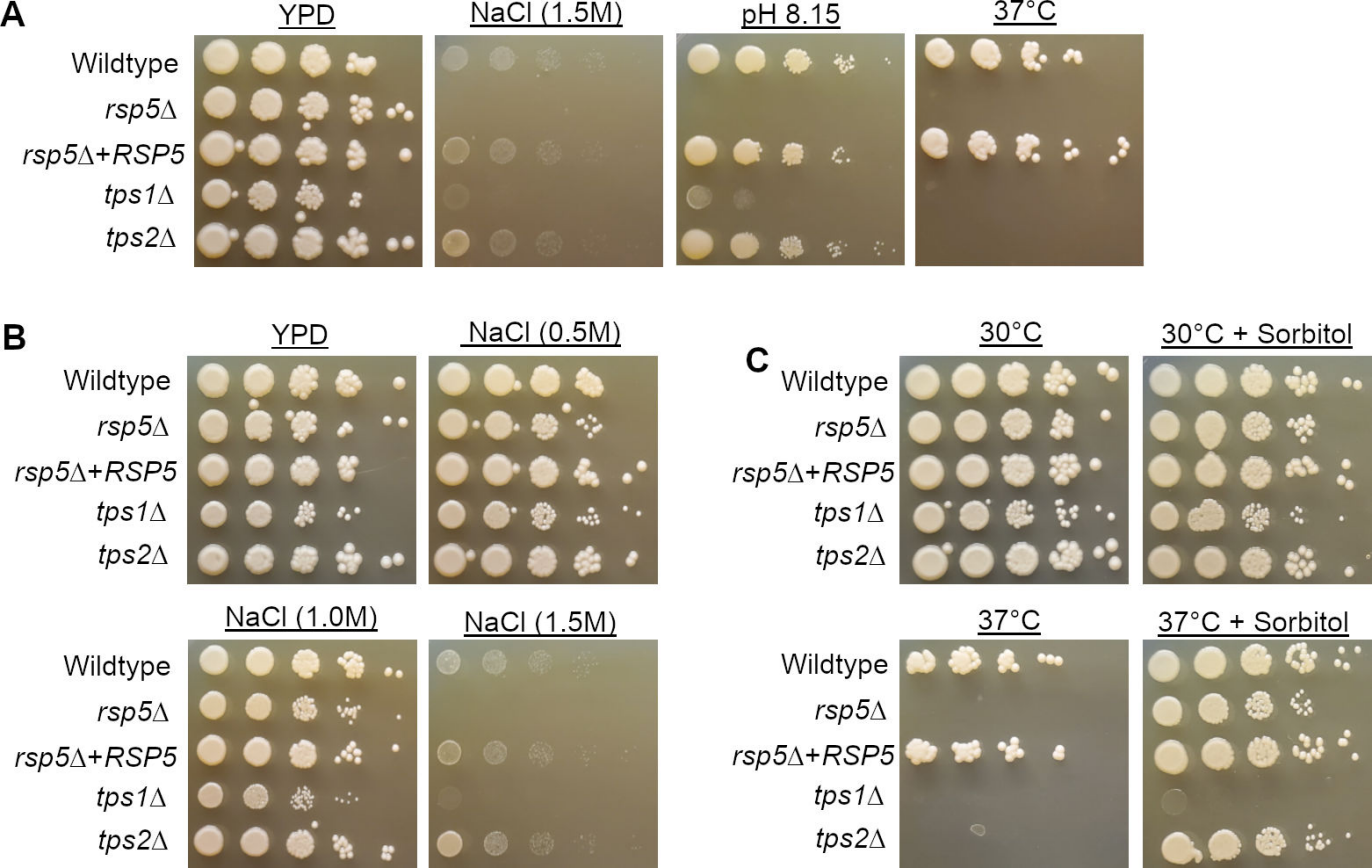
Rsp5-dependent temperature sensitivity is partially due to osmotic stress tolerance. (**A**) *C. neoformans rsp5*∆ and *tps1*∆ mutants have impaired growth under high salinity (NaCl 1.5 M), alkaline (pH 8.15), and high temperature (37°C) stress conditions, whereas the *tps2*∆ mutant only has a temperature-sensitive growth defect. (**B**) The temperature sensitivity of Rsp5 increases with a dose-dependent increase in NaCl, consistent with a model of osmotic stress. (**C**) The temperature-sensitive growth defect at 37°C for the *C. neoformans rsp5*∆ strain is partially restored by the addition of the osmotic stabilizer sorbitol (1 M). For (**A–C**), spot dilution assays performed by normalizing all strains to OD_600_ of 1.0, followed by serial 10× dilutions prior to pinning onto YPD plates with the indicated additive. Plates were then incubated at either 30°C or 37°C for 72 h. Representative images shown from three separate biological replicates.

*C. neoformans rsp5*∆, *tps1*∆, and *tps2*∆ mutant strains are all thermosensitive at human physiologic temperature (37°C), and all correspondingly have known reduced virulence in animal models (15, 18). However, only *rsp5*∆ and *tps1*∆ mutants have clear growth defects under osmotic and alkaline stress (Fig. 4A). The overlapping phenotypes with effectors that have established roles in *C. neoformans* stress tolerance may also provide insight into the molecular mechanisms of Rsp5-dependent stress adaptation. For example, the phenotype of impaired growth under high temperature for trehalose biosynthesis mutants is in part due to impaired osmotic tolerance. Accordingly, the addition of an osmotic stabilizer such as sorbitol to the growth medium restores thermotolerance to the *C. neoformans tps1∆ and tps2*∆ strains (18). Similarly, we also observed that for the *C. neoformans rsp5*∆ mutant, the NaCl growth defect occurs in a dose-dependent manner consistent with NaCl acting as an osmotic stressor (Fig. 4B). Therefore, we hypothesized that supplementation with sorbitol would also be able to restore growth of the *C. neoformans rsp5*∆ strain at elevated temperatures. Indeed, we found that the addition of exogenous sorbitol was sufficient to restore growth of the *rsp5*∆ strain at the elevated human physiologic temperature of 37°C (Fig. 4C). This discovery of an additional mechanism of Rsp5-dependent tolerance to temperature stress likely reflects the pleiotropic and multi-targeted nature of Rsp5 (Fig. 1A).

## DISCUSSION

*C. neoformans* utilizes the E3-ubiquitin ligase Rsp5 to adapt to a range of host-derived stressors, including high salinity, pH, and elevated temperature. By comparing the differences in ubiquitinated protein substrates between high salinity and alkaline stress conditions, we describe that Rsp5 ubiquitinates a core set of substrates in response to general cell stress in addition to ubiquitination of stress condition-specific substrates. The high salinity stress-specific Rsp5 substrates include trehalose biosynthesis enzymes, a *C. neoformans* stress response pathway with known roles in temperature sensitivity. Thus, we describe for the first time in a pathogenic fungus that ubiquitination regulates the trehalose biosynthesis pathway in response to stress conditions and identify an additional mechanism potentially underlying Rsp5-dependent temperature tolerance. Together with previous studies demonstrating Rsp5-dependent activation of distinct signaling cascades for temperature (Mpk1/Pkc1) (15) and pH (Rim101) (20) tolerance, this finding highlights the central and multi-targeted role of Rsp5 in *C. neoformans* stress response.

However, the mechanism by which Rsp5 integrates distinct environmental cues to coordinate context-specific downstream adaptations remains unknown. There are several potential alternative models that should be investigated. First, under specific stress conditions, the subcellular localization of Rsp5 may be altered and thereby place it in the proximity of relevant substrates that are then differentially ubiquitinated. Second, Rsp5 may retain the same substrate affinity in different stress conditions, but the relative abundance of its substrates is increased either transcriptionally or translationally during stress, thereby resulting in differential ubiquitination. Either of these alternative hypotheses could be further influenced by changes in Rsp5-adaptor proteins that coordinate the interaction between Rsp5 and many of its substrates. Further understanding of how Rsp5 responds to distinct stressors is likely to yield mechanistic insight into critical *C. neoformans* stress response pathways.

One of the most important ways in which ubiquitin ligases control substrate specificity is the use of differentially targeted adaptor proteins. For example, the arrestin-like Ali1 protein directs enzymes involved in fatty acid synthesis to sites of rapid cell membrane expansion during budding (28). A distinct arrestin domain-containing protein, Ali2, mediates a separate subset of Rsp5 interactions with target proteins involved in alkaline stress response (15). Despite demonstrating that *C. neoformans* Rsp5 is required for optimal trehalose biosynthesis in response to high temperature stress, future studies will be necessary to elucidate how Rsp5 interacts with components of the trehalose biosynthesis pathway and whether additional adaptor proteins support those interactions. It is possible that Tps1 and Tps2 directly bind to Rsp5 prior to their ubiquitination. These E3-ubiquitin ligase-target protein interactions are often facilitated by internal protein sequences such as the PXY motif required for Rsp5 interactions with its targets. We note that Tps1 has one canonical Rsp5 binding PXY motif. This suggests that Rsp5-Tps1 interactions may occur independently of adaptor proteins. In contrast, Tps2 does not contain any such motifs. Interestingly, our group has previously identified Tps2 as physically interacting with the Rsp5 adaptor protein Ali1 (15, 28), suggesting a model for the Rsp5-Tps2 interaction through this adaptor protein.

A limitation of this study is the lack of direct evidence of interaction between Rsp5 and Tps1 or Tps2. We demonstrated by direct measurement of Tps1 and Tps2 that Rsp5-dependent ubiquitination does not alter the total abundance of these substrates at either permissive or stress conditions (Fig. 2), and loss of Rsp5 actually increases *TPS1* and *TPS2* gene expression (Fig. 3F). These results are not consistent with a simple model in which Rsp5-mediated ubiquitination of target proteins results in their accelerated proteasomal degradation. Investigations of Rsp5-dependent ubiquitination in other model organisms suggest different hypotheses. For example, Rsp5-dependent ubiquitination can result in changes in the subcellular localization of its target proteins by differential vesicular sorting (29). Alternatively, the Rsp5-dependent changes in trehalose levels may be indirect, either by Rsp5-mediated regulation of an additional distinct E3 ligase or a cumulative effect involving a network of other cellular metabolism enzymes that have yet to be characterized. Moreover, downstream of the ubiquitination event, the impact of Rsp5-dependent ubiquitination of Tps1 and Tps2 needs to be further elucidated. As noted above, the lack of glycogen accumulation in the *rsp5*∆ mutant strain (Fig. 3E) implies some residual function of Tps1. This result highlights that while there is overlap between the two pathways, Rsp5-dependent ubiquitination and trehalose biosynthesis likely also have some non-overlapping functions. In the case of Rsp5, this may be attributed to alternative substrates (15, 20) and, in the case of Tps1, potentially due to the impact of uncharacterized secondary metabolites.

For both enzymes in the trehalose biosynthesis pathway that are ubiquitinated by Rsp5, we identified the ubiquitination event to occur on a lysine residue located in a basidiomycete-specific region of the protein sequence. *C. neoformans* is in the basidiomycete fungal phylum, which also includes plant fungal pathogens such as the maize pathogen *Ustilago maydis* and the rice blast fungus *Magnaporthe oryzae*, but which is divergent from ascomycete fungi such as *Saccharomyces cerevisiae*. This suggests a hypothesis that in basidiomycete fungi, such as *C. neoformans,* trehalose biosynthesis enzymes have undergone selective pressure to fine-tune the post-translational regulation of the enzymes rather than selection to alter enzyme function or specificity. Future studies will be needed to test whether the relationship between Rsp5 and trehalose biosynthesis has undergone convergent evolution in other fungi, and whether similar patterns have occurred in other Rsp5 substrates.

Here, we demonstrate that the pleiotropic nature of the E3-ligase Rsp5 enzyme can facilitate *C. neoformans* stress adaptation coordinated to specific environmental cues. One of these stressor-specific mechanisms is through upregulation of trehalose biosynthesis. An improved understanding of the cross-talk between these key regulators of *C. neoformans* stress response will help us to better understand how this environmental yeast becomes an opportunistic pathogen.

## MATERIALS AND METHODS

### Strains, media, and growth conditions

All strains used in this study were created in the *C. neoformans* var *grubii* H99 background (Table 2) (30). The *C. neoformans tps1*∆ and *tps2*∆ strains were provided as gifts from the lab of John R. Perfect and validated by presence-absence PCR with the following primer pairs: Tps1_Fwd (ATGCACATGGGCGGAACATA) with Tps1_Rev (GCCAGTTCCGACTAAAGCCT) and Tps2_Fwd (GCGGATCAGAAAGAGATTGC) with Tps2_Rev (CTTCTGATAGGCCAGCCAAG). Strains were recovered from glycerol stocks stored at −80°C onto solid YPD media (1% yeast extract, 2% peptone, 2% dextrose, and 2% agar) at 30°C. For each experiment, the strains were grown in liquid YPD media at 30°C in a shaking incubator at 150 rpms unless otherwise stated. Liquid YPD media were maintained at pH 5.5–6.0 unless otherwise indicated. For alkaline stress media, YPD media were buffered with 150 mM HEPES and then adjusted to pH 8.15 with potassium hydroxide. This pH, which is slightly more alkaline than the host pH (7.4), was empirically chosen to best visualize and distinguish cellular phenotypic differences between acidic and alkaline pH (5, 20). For high salinity stress media, the indicated concentration of sodium chloride was added to each medium condition.

**TABLE 2:** *C. neoformans* strains used in this study

Strain name/number	Genotype	Reference/source
H99	*MAT*α	(30)
MDP01	*MAT*α *rsp5*Δ::*NAT*	(15)
MDP26	*MAT*α *rsp5*Δ::*NAT* + *RSP5*	(15)
Tps1∆	*MAT*α *tps1*Δ::*NAT*	(19)
Tps2∆	*MAT*α *tps2*Δ::*NAT*	Gift from John R. Perfect

### Proteomics and differential ubiquitination determination

To quantify changes in total protein abundance after the osmotic stress of high salinity, after 18 h overnight culture, the indicated *C. neoformans* strains were diluted to an OD_600_ of 3.0 in 5 mL of YPD buffered to pH 4.0 with or without the addition of 1.5 M sodium chloride (NaCl). Strains were incubated at 30°C for 1 h prior to protein harvest. Protein harvest was performed by washing the cell pellet with distilled water twice, then resuspending in urea lysis buffer (8 M urea and 50 mM NH_2_HCO_3_) supplemented with cOmplete mini, EDTA-free protease inhibitor (Roche), Pierce phosphatase-inhibitor mini tablets (Thermo Scientific), and 1 mM phenylmethylsulfonyl fluoride (PMSF). Cells were then lysed by bead beating, resuspended in a total of 1.2 mL of the lysis buffer, and total protein quantified using a bicinchonic assay (BCA). All samples were normalized to a protein concentration of 1 μg/mL prior to protein quantification by liquid chromatography tandem mass-spectrometry (LC-MS/MS), as previously described (15, 28).

LC-MS/MS analysis was performed with the assistance of the Duke Proteomics and Metabolomics Core Facility, as previously described (15, 28). In brief, following protein solubilization in 8 M urea/50 mM ammonium bicarbonate, samples were brought to 5% SDS and reduced with 10 mM dithiothreitol for 15 min at 32°C, alkylated with 25 mM iodoacetamide for 20 min at room temperature, then trypsin digested with sequencing grade trypsin (Promega) on an STrap Micro (Protify) cartridge using manufacturer-recommended protocols.

Quantitative LC/MS/MS was performed on 250 ng of sample using an EvoSep One UPLC coupled to a Thermo Orbitrap Astral high-resolution accurate mass tandem mass spectrometer (Thermo). Briefly, each sample loaded EvoTip was eluted onto a 1.5 µm EvoSep 150 μm ID × 8 cm performance (EvoSep) column using the SPD60 gradient at 55°C. Data collection on the Orbitrap Astral mass spectrometer was performed in a data-independent acquisition (DIA) mode of acquisition with an *r* of 240,000 (at *m*/*z* 200) full MS scan from *m*/*z* 380–980 in the OT with a target AGC value of 4e5 ions. Fixed DIA windows of 4 *m*/*z* from *m*/*z* 380 to 980. DIA MS/MS scans were acquired in the Astral with a target AGC value of 5e4 and max fill time of 6 ms. HCD collision energy setting of 27% was used for all MS2 scans.

Raw data were imported into Spectronaut v20 (Biognosis), and MS/MS data were searched against a SwissProt *C. neoformans* H99 database (downloaded 2024). A library-free Direct DIA+ approach within Spectronaut was used to perform the database searches set at a maximum 1% peptide false discovery rate based on *q*-value calculations. Database search parameters included fixed modification on Cys (carbamidomethyl) with variable modification on Met (oxidation). Full trypsin enzyme rules were used along with 10 ppm mass tolerances on precursor ions and 20 ppm on product ions. Peptide homology was addressed using razor rules in which a peptide matched to multiple different proteins was exclusively assigned to the protein with the highest % sequence coverage. A MaxLFQ rollup strategy was deployed to roll up from the precursor level to the protein level (31). Determination of differentially ubiquitinated substrates was described previously for high salinity (NaCl 1.5M) and alkaline (pH 8.15) stress (15, 20). In brief, 1 mg of trypsin digested cell lysate was enriched using the PTMScan HS Ubiquitin/SUMO Remnant Motif (K-ε-GG) Kit (Cell Signaling Technologies) according to the manufacturer’s protocols (https://media.cellsignal.com/pdf/59322.pdf). Eluted peptides were analyzed using a nanoAcquity UPLC system (Waters Corp) coupled to a Thermo Orbitrap Fusion Lumos high-resolution accurate mass tandem mass spectrometer (Thermo). Peptides were trapped on a Symmetry C18 20 mm × 180 µm trapping column (5 μL/min at 99.9/0.1 [vol/vol] water/acetonitrile), after which the analytical separation was performed using a 1.8 µm Acquity HSS T3 C18 75 µm × 250 mm column (Waters Corp.), with a 90-min linear gradient of 5%–30% acetonitrile with 0.1% formic acid at a flow rate of 400 nanoliters/minute (nL/min) with a column temperature of 55°C. Data collection was performed in a data-dependent acquisition (DDA) mode of acquisition with an *r* of 120,000 (at *m*/*z* 200) full MS scan from *m*/*z* 375–1,500 with a target AGC value of 4e5 ions. MS/MS scans were acquired in the linear ion trap in “rapid” mode with a target AGC value of 1e5 and a max fill time of 100 ms.

Data were imported into Proteome Discoverer 3.0 (Thermo Scientific Inc.), and the MS/MS data were searched against the SwissProt *C. neoformans* H99 database (downloaded in 2023) and an equal number of reversed-sequence “decoys” for false discovery rate determination. Sequest (v 3.0, Thermo PD) was utilized to produce fragment ion spectra and perform the database searches. Database search parameters included fixed modification on Cys (carbamidomethyl) and variable modification on Met (oxidation) and Lys (-GG). Search tolerances were 2 ppm precursor and 0.8 Da product ion with full trypsin enzyme rules. Peptide Validator and Protein FDR Validator nodes in Proteome Discoverer were used to annotate the data at a maximum 1% protein false discovery rate based on q-value calculations. In this study, the set of differentially ubiquitinated substrates with at least 5-fold enrichment in the wild type compared to the *rsp5*∆ strain was used for further analysis. All mass spectrometry data have been uploaded to ProteomeXchange.

### Gene ontology analysis

Gene ontology analysis was performed using the FungiFun3 webserver (32). Overrepresentation analysis was performed using gene ontology terms with *C. neoformans* var. grubii serotype A (strain H99/ATCC208821/CBS10515/FGSC9487) specified as reference species. Unless otherwise specified, default parameters were used.

### Phylogenetic analysis

*C. neoformans TPS2* (*CNAG_03765*) homologs were identified through the FungiDB (33) platform by BlastP search with an E-value cutoff of 0.0001. The search was restricted to the following set of manually curated representative fungi: *Aspergillus fumigatus* Af293*, Aspergillus nidulans* FGSC A4*, Candida albicans* SC5314*, Candida auris* strain B8441*, Coccidioides immitis* RS*, Coccidioides posadasii* strain Silveira*, Cryptococcus* cf*. gattii MF34, Cryptococcus gattii* VGII R265*, C. neoformans* var. grubii H99*, C. neoformans* var. neoformans JEC21*, Histoplasma capsulatum* G217B*, Kwoniella mangroviensis* CBS 8507*, Magnaporthiopsis poae* ATCC 64411*, Malassezia restricta* KCTC 27527*, Mucor circinelloides* 1006PhL*, Pneumocystis jirovecii* SE8*, Saccharomyces cerevisiae* S288C*, Tremella mesenterica* DSM 1558*, Trichosporon asahii* var. asahii CBS 2479*,* and *Ustilago maydis* 521. This resulted in the identification of 64 proteins, which included the related but distinct protein Tps1. To restrict the phylogeny to Tps2 homologs, the initial set of results was filtered to include only those with >40% sequence identity to the CNAG_03765 reference sequence. Multisequence alignment was then performed with default parameters using the Clustal algorithm on the EMBL-EBI webserver (34). The corresponding phylogenetic tree alignment was visualized in R using the ggtree package (35). Multisequence alignment was visualized using Jalview (36).

### Stress response assays

*C. neoformans* stress response phenotypes were assayed by spot-dilution assay, as described previously (37). In brief, overnight cultures of the indicated strains of *C. neoformans* were diluted to an OD_600_ of 1.0 prior to making 5 serial 10× dilutions in 200 μL of YPD in 96-well plate format. They were then spotted in replicate onto the indicated plate with a standardized volume using a metal-pronged pinner. Plates were incubated in a temperature-controlled incubator at 30°C unless otherwise indicated.

### Trehalose quantification and glycogen staining

Intracellular trehalose concentration was measured as previously described in *S. cerevisiae, A. fumigatus, and C. neoformans* (17, 24–26). After the indicated stress conditions, cell concentration was determined using a hemocytometer. A standardized volume for the same total number of cells was centrifuged at room temperature at 3,000 × *g* for 5 min and washed once with sterile deionized H_2_O. Then, the supernatant was removed, and the remaining cell pellet was placed in a lyophilizer overnight. Cells were then disrupted by placing 3 mm glass beads into the 15 mL culture tube with the dried cell pellet, followed by vortexing vigorously for 30 s. Cellular debris was then resuspended in 200 μL of sterile PBS prior to transfer to a microcentrifuge tube. The cellular debris was then centrifuged in a tabletop centrifuge at 13,000 rpm for 2 min, and the supernatant was transferred to a new sterile microcentrifuge tube. Each sample was then treated overnight at 37°C with trehalase by combining 30 μL of sample, 30 μL of sodium citrate 0.1 M, and 10 μL of porcine trehalase at 0.00033 U/mL working concentration (Millipore Sigma; T8778). A vehicle control was prepared in parallel for each sample with no added trehalase. Finally, the total glucose resulting from each sample was quantified using a colorimetric glucose detection assay per the manufacturer’s protocol in microplate format (Millipore Sigma; GAGO20). Total trehalose was calculated as follows: $\frac{{\left[glucose\right]}_{trehalase\;treated}\;-\;{\left[glucose\right]}_{vehicle\;treated}}{culture\;concentration}$. Culture concentration was determined by a hemocytometer or measurement of OD_600_ as indicated.

### *TPS1* and *TPS2* gene expression quantification and analysis

*TPS1* and *TPS2* gene expression analysis was performed by qRT-PCR using the ∆∆*C_t_* method. *C. neoformans* mRNA was harvested as previously described (20). In brief, after the indicated stress condition, *C. neoformans* cultures were harvested by centrifugation at 3,000 × *g* for 5 min prior to removing the supernatant. Cell pellets were then flash frozen on dry ice and lyophilized overnight prior to proceeding with mRNA extraction using the Qiagen RNeasy Plant Minikit (Qiagen). Total mRNA was normalized to 0.5 μg in 10 μL, which was used as input for cDNA synthesis using the iScript cDNA synthesis kit (BioRad). Primers for *TPS1* (AA6130 CGGCAAAGATATCCCCATGC and AA6131 TCGCCGGTGATCATAGAACG) and *TPS2* (AA6132 TGCCTCCCTAAGAGGAGAGA and AA6133 GGTCTCGAAGAGCCTGCATT) were designed with the forward primer overlapping an exon junction to be specific for the mRNA template rather than genomic DNA. The housekeeping gene *GPD1* was amplified with previously described primers (AA301 and AA302) (38) and used to calculate relative expression by the ∆∆*C*_*t*_ method.

## Data Availability

Data from the proteomics experiments generated in this study are available in supplemental material and in the ProteomeXChange database (PXD071777). All other data are available upon request.

## SUPPLEMENTAL MATERIAL

Supplemental figures msphere.00212-26-s0001.pdf Fig. S1 and S2.

Supplemental tables msphere.00212-26-s0002.xlsx Tables S1 and S2.

ASM does not own the copyrights to Supplemental Material that may be linked to, or accessed through, an article. The authors have granted ASM a non-exclusive, world-wide license to publish the Supplemental Material files. Please contact the corresponding author directly for reuse.
