# Supplementary material for: Interaction between Rsp5-dependent ubiquitination and trehalose production during Cryptococcus neoformans temperature stress adaptation
Source: mSphere. 2026 May 20;11(6):e00212-26. doi: 10.1128/msphere.00212-26 (PMC13317204; doi:10.1128/msphere.00212-26)
Supplement: Supplemental figures — Fig. S1 and S2. [file msphere.00212-26-s0001.pdf]

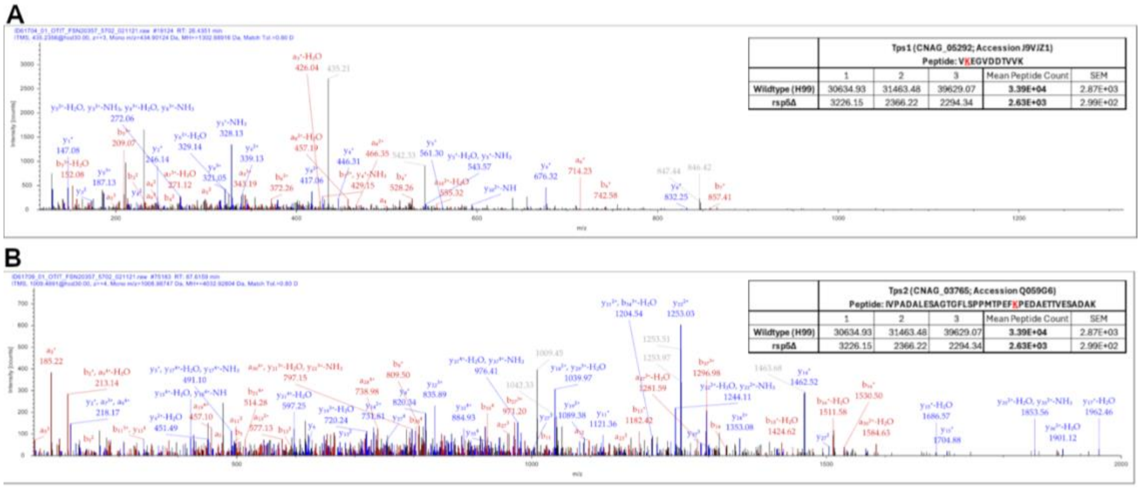

	Tps1 (CNAG_05292; Accession J9VJZ1) Peptide: VKEGVDDTVVK				
	1	2	3	Mean Peptide Count	SEM
**Wildtype (H99)**	30634.93	31463.48	39629.07	**3.39E+04**	2.87E+03
**rsp5Δ**	3226.15	2366.22	2294.34	**2.63E+03**	2.99E+02

	Tps2 (CNAG_03765; Accession Q059G6) Peptide: IVPADALESAGTGFLSPPMTPEFKPEDAETTVESADAK				
	1	2	3	Mean Peptide Count	SEM
**Wildtype (H99)**	30634.93	31463.48	39629.07	**3.39E+04**	2.87E+03
**rsp5Δ**	3226.15	2366.22	2294.34	**2.63E+03**	2.99E+02

Supplemental Figure S1. Representative mass spectrometry spectra of ubiquitinated peptides mapped to Tps1 and Tps2. MS/MS spectrum of Tps1 (A) and Tps2 (B) following HCD fragmentation and product ion detection in the linear ion trap of a Fusion Lumos high resolution mass spectrometer. Y and B fragment ion series detected within FDR controlled Mascot database searches are highlighted. The ubiquitinated lysine residues (K78 on Tps1 and K228 on Tps2) are highlighted in the text of superimposed tables in red. The normalized peptide counts between the *C. neoformans* wildtype and *rsp5*Δ conditions are shown from three independent biological replicates with mean and standard error of the mean.

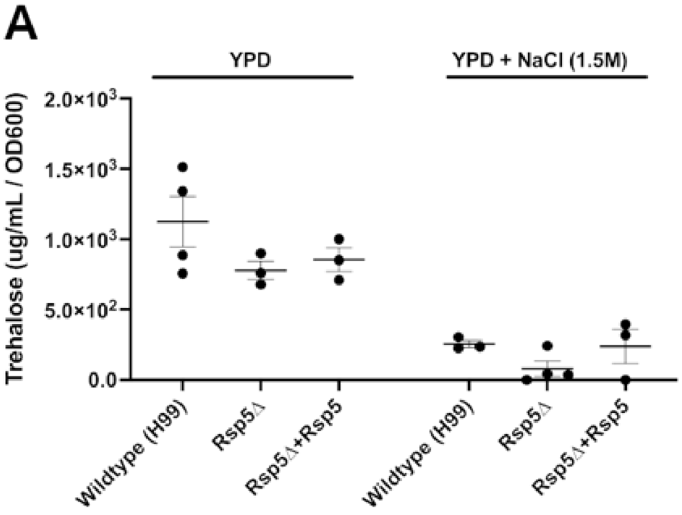

Supplemental Figure S2. ***C. neoformans*** **has significantly reduced intracellular trehalose after exposure to NaCl (1.5M) stress.** *C. neoformans* wildtype, *rsp5*Δ, or *rsp5*Δ*+RSP5* was incubated in liquid media (YPD) with and without additional supplementation of NaCl (1.5M). Trehalose was quantified after 8 hours of incubation and normalized to culture concentration by measurement of OD_{600} at the end time-point. Mean and standard error of the mean are plotted from 3-4 biological replicates per condition. Samples below the dynamic range of the standard curve were assumed to have a concentration of 0μg/mL. Statistical analysis was performed by two-way ANOVA with Tukey's post-hoc test. The variable of NaCl supplementation explained 68.96% of the variation ($p < 0.001$); however, no individual post-hoc comparisons reached statistical significance at a threshold of $p < 0.05$.
